# Arctic, Antarctic, and temperate green algae *Zygnema* spp. under UV-B stress: vegetative cells perform better than pre-akinetes

**DOI:** 10.1007/s00709-018-1225-1

**Published:** 2018-02-22

**Authors:** Andreas Holzinger, Andreas Albert, Siegfried Aigner, Jenny Uhl, Philippe Schmitt-Kopplin, Kateřina Trumhová, Martina Pichrtová

**Affiliations:** 1 Department of Botany, Functional Plant Biology, University of Innsbruck, Sternwartestraße 15, 6020 Innsbruck, Austria; 2 Research Unit Environmental Simulation, Helmholtz Zentrum München – Deutsches Forschungszentrum für Gesundheit und Umwelt GmbH, Ingolstaedter Landstr. 1, 85764 Neuherberg, Germany; 3 Research Unit Analytical BioGeoChemistry, Helmholtz Zentrum München – Deutsches Forschungszentrum für Gesundheit und Umwelt GmbH, Ingolstaedter Landstr. 1, 85764 Neuherberg, Germany; 4 Faculty of Science, Department of Botany, Charles University, Benátská 2, 128 01 Prague, Czech Republic

**Keywords:** UV-A, UV-B, UV simulation, Green algae, Ultrastructure, Metabolomics

## Abstract

Species of *Zygnema* form macroscopically visible mats in polar and temperate terrestrial habitats, where they are exposed to environmental stresses. Three previously characterized isolates (Arctic *Zygnema* sp. B, Antarctic *Zygnema* sp. C, and temperate *Zygnema* sp. S) were tested for their tolerance to experimental UV radiation. Samples of young vegetative cells (1 month old) and pre-akinetes (6 months old) were exposed to photosynthetically active radiation (PAR, 400–700 nm, 400 μmol photons m^−2^ s^−1^) in combination with experimental UV-A (315–400 nm, 5.7 W m^−2^, no UV-B), designated as PA, or UV-A (10.1 W m^−2^) + UV-B (280–315 nm, 1.0 W m^−2^), designated as PAB. The experimental period lasted for 74 h; the radiation period was 16 h PAR/UV-A per day, or with additional UV-B for 14 h per day. The effective quantum yield, generally lower in pre-akinetes, was mostly reduced during the UV treatment, and recovery was significantly higher in young vegetative cells vs. pre-akinetes during the experiment. Analysis of the deepoxidation state of the xanthophyll-cycle pigments revealed a statistically significant (*p* < 0.05) increase in *Zygnema* spp. C and S. The content of UV-absorbing phenolic compounds was significantly higher (*p* < 0.05) in young vegetative cells compared to pre-akinetes. In young vegetative *Zygnema* sp. S, these phenolic compounds significantly increased (*p* < 0.05) upon PA and PAB. Transmission electron microscopy showed an intact ultrastructure with massive starch accumulations at the pyrenoids under PA and PAB. A possible increase in electron-dense bodies in PAB-treated cells and the occurrence of cubic membranes in the chloroplasts are likely protection strategies. Metabolite profiling by non-targeted RP-UHPLC-qToF-MS allowed a clear separation of the strains, but could not detect changes due to the PA and PAB treatments. Six hundred seventeen distinct molecular masses were detected, of which around 200 could be annotated from databases. These results indicate that young vegetative cells can adapt better to the experimental UV-B stress than pre-akinetes.

**Electronic supplementary material:**

The online version of this article (10.1007/s00709-018-1225-1) contains supplementary material, which is available to authorized users.

## Introduction

The effects of UV radiation on green algae have been studied extensively (reviewed by, e.g., Holzinger and Lütz 2006; Karsten and Holzinger 2014; Holzinger and Pichrtová 2016), mainly after the detection of stratospheric ozone holes over the polar regions, increasing UV-B radiation. This could lead to destructive effects on chloroplasts and DNA, which in turn would influence algal development and distribution. Different avoidance and protection mechanisms have been described, particularly in groups that live in terrestrial habitats.

Studies have focused on UV shielding and protecting substances, which vary widely in different groups of green algae. In Zygnematophycean green algae, unusual phenolic compounds with UV-absorbing capacities have been found in *Spirogyra* sp. and *Zygnema* sp. (e.g., Nishizawa et al. 1985; Cannell et al. 1988; Pichrtová et al. 2013). These phenolic substances may also absorb in the visible waveband, such as the red vacuolar pigment in *Zygogonium ericetorum*, a glycosylated derivative of gallic acid, complexed with ferric iron (e.g., Aigner et al. 2013; Herburger et al. 2016). In the ice-algae *Ancylonema nordenskiöldii* (Remias et al. 2012a) and *Mesotaenium berggrenii*, purple to brown visible and UV-absorbing compounds were found, the latter characterized as purpurogallin-derived secondary pigment (Remias et al. 2012b). Several, particularly chlorophytic green algae contain different UV-absorbing compounds, such as mycosporine-like amino acids (MAAs; e.g., Karsten et al. 2007; Hartmann et al. 2016). MAAs were also found in basal streptophytic green algae, where they had slightly different absorption spectra with a peak at 324 nm (Kitzing et al. 2014). Other chlorophytes are protected by secondary carotenoids, pigments of the astaxanthin family, giving them a red appearance (e.g., Remias et al. 2005). Because Zygnematophyceae possess neither MAAs nor secondary carotenoids, we focused our investigations on phenolic compounds.

Several studies have investigated the effects of UV radiation on Zygnematophycean green algae (e.g., Meindl and Lütz 1996; Lütz et al. 1997; Holzinger et al. 2009; Germ et al. 2009; Pichrtová et al. 2013; Stamenković and Hanelt 2014; Prieto-Amador 2016; Stamenković and Hanelt 2017). Pichrtová et al. (2013) investigated the changes in phenolic compounds in three species of *Zygnema* from either Arctic or Antarctic habitats. These species, *Zygnema* sp. B (also included in the present study), *Zygnema* sp. G, and *Zygnema* sp. E, all showed a significant increase in total phenolic compounds (Pichrtová et al. 2013). For the present study, we selected the Antarctic *Zygnema* sp. C, which an *rbc*L analysis proved to be identical to the previously investigated *Zygnema* sp. E (Pichrtová et al. 2014). According to Stancheva et al. (2012), the genus *Zygnema* is divided into two major clades. The strains investigated here all belong to the same clade, where *Zygnema* sp. B and C are closely related to *Z. irregulare* (Pichrtová et al. 2014) and *Zygnema* sp. S to *Z. circumcarinatum* (Herburger et al. 2015). All three strains were previously characterized concerning their physiological and ultrastructural parameters (Kaplan et al. 2013; Pichrtová et al. 2013, 2014; Herburger et al. 2015). In *Zygnema* sp. S, hyperspectral characterization was preformed that allowed to acquire a total absorption spectrum in the range of 400–900 nm (Holzinger et al. 2016).

The possibilities in UV simulation under experimental conditions are limited. In cultured *Zygnema* spp., we used previously a UV simulation that was described as a predominantly UV-A treatment (Pichrtová et al. 2013). Therefore, the “sun-simulation system” at the Helmholtz Center in Munich is used, which creates realistic PAR to UV conditions (Remias et al. 2010; Hartmann et al. 2015). Hartmann et al. (2015) exposed the chlorophyte green algae *Pseudomuriella engadiensis* and *Coelastrella terrestris* in the same sun-simulation device used in the present study; by exposing the cells to 13.4 W m^−2^ UV-A and UV-B up to 2.8 W m^−2^, they found an enhancement of some primary metabolites, mainly aromatic amino acids, nucleic bases, and nucleosides (Hartmann et al. 2015). In a study by Remias et al. (2010) applying this sun simulator, the chlorophytic snow alga *Chlamydomonas nivalis* and a terrestrial alga from a polar habitat were investigated by relatively high PAR of 724 μmol photons m^−2^ s^−1^ that was combined with UV-A values of 15.9 W m^−2^ and UV-B values of up to 1.43 W m^−2^ (Remias et al. 2010). A study on different strains of the desmid *Cosmarium* used 700 μmol photons m^−2^ s^−1^ in combination with 27.5 W m^−2^ UV-A or 28.7 W m^−2^ UV-A and 0.89 W m^−2^ UV-B (Stamenković and Hanelt 2014). Arctic *Zygnema* sp. were even exposed to gamma radiation (Choi et al. 2015), which resulted in drastic changes of photosynthesis-related proteins; however, the potential for repair was shown by upregulation of proteins related to DNA repair, quinone oxigoreductase, cytoskeleton, and cell wall biogenesis (Choi et al. 2015).

The present study exposed *Zygnema* species of (A) different culture ages, i.e., young vegetative cells and mature pre-akinetes, to realistic simulated UV conditions in a sun-simulation chamber. We hypothesized that older pre-akinetes could tolerate UV stress better. This hypothesis was mainly driven by the observations that pre-akinetes showed generally better stress tolerance, e.g., to desiccation stress (e.g., Pichrtová et al. 2014) or to freezing during winter (Pichrtová et al. 2016a). A recent transcriptomic study in *Zygnema cricumcarinatum* (*Zygnema* sp. S) revealed that upon desiccation stress, about 1200 transcripts were up- or downregulated in young vegetative cells, while in pre-akinetes, only 400 transcripts were regulated (Rippin et al. 2017). This was attributed to a hardening process, making less regulation necessary. The comparison between young vegetative cells and pre-akinetes concerning UV tolerance was not yet studied using an experimental approach, as previously either field-collected samples of pre-akinete stage (Holzinger et al. 2009) or young cultured material of *Zygnema* sp. (Pichrtová et al. 2013; Prieto-Amador 2016) was investigated.

Moreover, the present study investigated *Zygnema* species of (B) different geographic origins, i.e., the Arctic (*Zygnema* sp. B), Antarctic (*Zygnema* sp. C), and a temperate isolate (*Zygnema* sp. S). As the polar strains are exposed to milder UV scenarios in their natural habitat in combination with the permanent radiation of a polar day, we hypothesized that they might show differences in tolerating the experimental UV exposure. The significance of different geographic distribution in UV tolerance has been investigated in different strains of *Cosmarium* sp. (Stamenković and Hanelt 2014). Untreated and UV-exposed samples were investigated for changes in primary pigments and phenolic compounds, using a metabolomics approach, to determine if there are differences among the individual strains, the culture age, and the UV exposures. The structural changes were investigated by light- and transmission electron microscopy.

## Material and methods

### Algal strains

For the present study, three different strains of *Zygnema* with different geographical origins were used: a strain *Zygnema* sp. S (Culture collection Göttingen, SAG 2419, previously isolated from a sandbank of the Saalach River, Salzburg, Austria, at about 440 m a.s.l., Herburger et al. 2015); an Arctic isolate from Svalbard, *Zygnema* sp. B (Culture Collection of Autotrophic Organisms in Trebon, Czech Republic CCALA, www.butbn.cas.cz/ccala/index.php; isolated on Svalbard in 2010, accession number CCALA 976); and the Antarctic isolate *Zygnema* sp. C (CCALA 880), previously isolated from James Ross Island. The algae were cultured on Bold’s Basal Medium (BBM) solidified with 1.5% agar. The cultures were maintained under either continuous illumination or a light-dark cycle of 16:8 h at 15 °C at ~ 38 μmol photons m^−2^ s^−1^. For the experiments, either young cultures (1 month) or 6-month-old cultures consisting of well-developed pre-akinetes were used (Pichrtová et al. 2014).

### Experimental UV simulation

For the UV treatments, the algae were placed in the sun simulator at the Helmholtz Center Munich, to study the algae’s response under a simulated natural photophysiological environment. In the sun simulator, a combination of four lamp types (metal halide lamps: Osram Powerstar HQI-TS 400W/D, quartz halogen lamps: Osram Haloline 500W, blue fluorescent tubes: Philips TL-D 36W/BLUE, and UV-B fluorescent tubes: Philips TL 40W/12) was used to obtain a natural balance of simulated global radiation throughout the UV to infrared spectrum. The short-wave cut-off was achieved by selected soda lime and acrylic glass filters. Detailed descriptions of the sun simulator facility were given by Döhring et al. (1996) and Thiel et al. (1996). The experimental period was 74 h. The radiation period lasted for 16 h per day, with 400 μmol m^−2^ s^−1^ PAR (400–700 nm) plus UV-A (315–400 nm)—this mimics the natural situation, where PAR is always combined with UV-A (designated as PA); UV-B radiation (280–315 nm) was added 1 h after the start of illumination and switched off 1 h before the dark phase, providing a total UV-B exposure of 14 h per day (designated as PAB). The duration of the light phase was chosen to simulate long summer days, as realistic for the temperate strain. The duration of the experiment was previously found to generate UV-induced changes in various algae exposed in the same sun simulator (Hartmann et al. 2015). The samples were harvested on the 4th day, 2 h after the onset of the UV-B exposure. The intensities of UV-A and UV-B radiation are shown in Table 1. The spectral composition during the experimental procedure is illustrated in Suppl. Fig. S1.

**Table 1:** Applied UV radiation during the experiment in the exposure chamber

	PA	PAB
UV-B	0	1.0 W m^-2^
UVBbe*	0	241 mW m^-2^
UV-A	5.7 W m^-2^	10.1 W m^-2^
PAR	400 μmol photons m^-2^ s^-1^ or all	

*Biologically effective UV-B. Plant action spectrum after Caldwell 1971, normalized at 300 nm

### Chlorophyll fluorescence

Effective quantum yield (*ϕ*_PSII_) measurements were performed with a PAM 2500 (Walz, Germany) on PA- and PAB-exposed cells during the experiment 2 h after switching on the UV-B lamp, as previously described (Pichrtová et al. 2014). For the measurements, the samples were removed from the exposure chamber for the shortest possible time (5 min or less).

### HPLC analysis of primary pigments and phenolics

HPLC analysis of primary pigments and phenolic compounds was performed with untreated samples (harvested prior to the experiment, 0) and with samples harvested at the end of the PA or PAB exposure. Vegetative and pre-akinete cells of *Zygnema* sp. C and *Zygnema* sp. S were used in three replicates each. For *Zygnema* sp. B, insufficient biomass was available to perform these analyses.

Freeze-dried material was ground with glass beads, using a laboratory mill (Tissuelyser II, Qiagen, Venlo, The Netherlands) at 30 Hz for 10 min and extracted as described by Aigner et al. (2013) with minor modifications. The powder was suspended in 1 ml methyl-tertbutylether (MTBE, Sigma-Aldrich, St. Louis, USA) containing 0.1% butylated hydroxytoluene (BHT, Sigma-Aldrich, St. Louis, USA) to prevent oxidation of pigments. Then, the extract was vortexed and sonicated for 15 min at 0 °C and the supernatant was removed; the sedimented material was again resuspended in 1.5 ml MTBE to assure quantitative extraction. Both MTBE extracts were combined, and then 2 ml of 20% methanol (*v*/*v*; Roth, Karlsruhe, Germany) was added to the material and shaken at 4 °C, and the samples were frozen overnight at − 20 °C. This extract was then centrifuged (1000*g*, 5 min) at 4 °C to support phase separation of the lipophilic supernatant (MTBE phase) and the hydrophilic lower (methanol) phase. The upper and the lower phases were separated, evaporated to dryness in a SpeedVac (SPD111V, Thermo Fisher Scientific, Waltham, USA), and then resuspended in 350 μl *N*,*N*-dimethylformamide (DMF, Scharlau, Sentmenat, Spain) and 350 μl of 50% methanol (*v*/*v*; HPLC grade, Roth, Karlsruhe, Germany), respectively. The extracts were centrifuged (15,000*g*, 45 min, 4 °C) prior to injection into the HPLC.

Primary pigments were quantitatively analyzed according to Remias et al. (2005) with minor modifications, on an Agilent Technologies 1100 system (Waldbronn, Germany), with a DAD-detector set at 440 nm for carotenoids and 662 nm for chlorophyll *a*. The column was a LiChroCART (C18, 100 × 4.6 mm, 5 μm, 120 A) column (Agilent, Waldbronn, Germany) at a flow rate of 1 ml min^−1^ using solvent A (acetonitrile:methanol = 74:6) and solvent B (methanol:hexane = 5:1). The system was started at 0% solvent B for 4 min, followed by a gradient to 100% solvent B from 4 to 9 min, which was maintained for 9 min, followed by a 5-min post-run with 100% solvent A. All solvents were HPLC grade. Pigment calibration and quantification were undertaken for ß-carotene and zeaxanthin with standards from Carbon 14 Centralen, Hørsholm, Denmark, while chlorophyll *a* was obtained from Sigma-Aldrich. All experimental manipulations were carried out in dim light at low temperatures. The phenolic pigments were analyzed from the hydrophilic phase in the same system and separated using a Phenomenex Synergi Polar-RP column (150 × 3.0 mm, 4 μm, 80 A; Aschaffenburg, Germany), protected with an RP-18 guard cartridge (20 × 4 mm I.D.) of the same material, at 25 °C with a flow rate of 0.3 ml min^−1^ and an injection volume of 25 μl.

Mobile phases are as follows: (A) water + 0.5% formic acid (*v*/*v*) and (B) methanol + 0.5% formic acid (*v*/*v*). The binary linear solvent gradient was as follows: start 0% B; 40 min: 100% B; followed by an 8-min post-run with 100% A. Whole absorbance spectra were recorded each second, and DAD detection wavelengths were 280 and 350 nm, respectively, after Aigner et al. (2013).

### Metabolic profiling of *Zygnema* strains

Samples of vegetative and pre-akinete cells of *Zygnema* spp. B, C, and S were taken before and after UV treatment, in triplicate. Algal material was transferred into NucleoSpin® Bead Tubes (Macherey-Nagel, Germany) and evaporated until dryness to calculate the dry weight. Cells were extracted with 500 μl 70% methanol (Chromasolv™, Sigma-Aldrich, Germany) in 30% purified water (*v*/*v*) using a Precellys® Homogenizer (Bertin Technologies, France) at around 4 °C and 2650*g* (3 times at 20 s). After centrifugation for 15 min at 4 °C and 20,800*g*, supernatants were removed and stored at − 80 °C for further analysis.

Metabolic analyses were performed using reversed=phase ultrahigh-performance liquid chromatography (UHPLC; Waters Acquity) coupled to a time-of-flight mass spectrometer (qToF–MS; Bruker Daltonik maXis) with positive ionization mode. The maXis qToF–MS provides a resolution of > 50,000 at m/z 400 and a mass accuracy < 2 ppm. All chemicals used were LC-MS grade (Chromasolv™), provided by Sigma-Aldrich, Germany.

Mobile phases containing (A) purified water with 0.1% formic acid (*v*/*v*) and (B) acetonitrile with 0.1% formic acid (*v*/*v*) were applied for chromatographic separation on a Waters Acquity BEH C_18_ column (dimensions 100 mm × 2.1 mm ID, 1.7 μm particle size) at 40 °C. A 10-min gradient was processed from 0 to 1.12 min with 0.5% B, followed by a continuous increase of B until 99.5% at 6.41 min and a stable highly non-polar plateau of 99.5% B until 10.01 min. Equilibration of the stationary phase was ensured by a pre-run time set to 2 min with 0.5% B. Samples were stored at 4 °C during the measurements. Five microliters of each sample extract was injected at a flow rate of 0.4 ml min^−1^. Mass spectra were acquired within a mass range of 100–1500 m/z at 2.0 Hz scan rate (for additional parameters see Suppl. Table S1).

Data were processed with Genedata Expressionist V10.5 (Genedata AG, Switzerland). To ensure quality of the spectra and reliability of the measurements over time, a certified standard (ESI-L Low Concentration Tuning Mix, Agilent Technologies, Germany) was injected in the mass spectrometer at the beginning of each run. The resulting peak in each total ion chromatogram (TIC) was used to create a verified chromatogram grid over all the data, and the resulting exact masses were used for calibration of MS spectra. After blank subtraction, the remaining sample peaks were integrated and isotopic clusters were assigned automatically. Masses only present in one sample were not taken into account. Therefore, 617 molecular masses were determined within the sample set, which were further analyzed statistically for their response to the UV treatments of the three *Zygnema* strains.

### Light- and transmission electron microscopy

Light microscopy was performed on 2.5% glutaraldehyde-fixed cells (see below) with an Olympus BX5 microscope equipped with an Olympus DP72 camera and QuickPhoto Camera 2.3 software.

For transmission electron microscopy, specimens of *Zygnema* spp. B, C, and S exposed to PA or PAB were fixed with a standard chemical fixation protocol according to Holzinger et al. (2009) with modifications. Briefly, cells were fixed in 2.5% glutaraldehyde at room temperature for 1.5 h, rinsed, and post-fixed in 1% OsO_4_ at 4 °C overnight; both fixatives were dissolved in 20 mM cacodylate buffer, pH 7. After dehydration in increasing ethanol steps, cells were embedded in modified Spurr’s resin and heat-polymerized. Ultrathin sections were counterstained with uranyl acetate and Reynold’s lead citrate and investigated in Zeiss LIBRA 120 transmission electron microscopes at 80 kV. Images were captured with a TRS 2k SSCCD camera and further processed using Adobe Photoshop software (Adobe Systems Inc., San José, CA, USA).

### Statistical evaluation of the data

The data for the phenolic concentrations as well as the deepoxidation state were evaluated using a three-way ANOVA analysis, with three factors “strain,” “UV treatment,” and “culture age” considered as factors with fixed effects. Differences between individual UV treatments were tested by one-way ANOVA analyses followed by Tukey’s post hoc tests, separately for each strain and culture age. Relative values of the effective quantum yield corresponding to the recovery rate of the initial values measured at the end of the experiment were also tested by three-way ANOVA, and additional two-way ANOVA analyses were performed for the individual strains separately. For all analyses the significance value was set as *p* < 0.05. The analyses were performed in Statistica 10 for Windows and PAST (Hammer et al. 2001). All results of statistical analyses are summarized in Suppl. Table S2.

Statistical evaluation of metabolomics data was performed using Genedata Expressionist V10.5 (Genedata AG, Switzerland). Data were first normalized to the sample dry weight and categorized according to *Zygnema* strain, UV treatment, culture age, and biological replicate. Applied N-Way ANOVA analyses including the factors strain type, culture age, and UV treatment did not give significance values of *p* < 0.06. Principal components analyses (PCAs) of covariances were performed based on relative contents, i.e., the peak area of a single peak in relation to the summed peaks in the spectra. Metabolite alignment was done using an adapted version of the MassTRIX webserver (Suhre and Schmitt-Kopplin 2008). The maximum error for annotated masses was set to 0.005 Da, and the possible appearance of sodium and formic acid adducts was taken into account.

## Results

### Changes in effective quantum yield

**Fig. 1:**
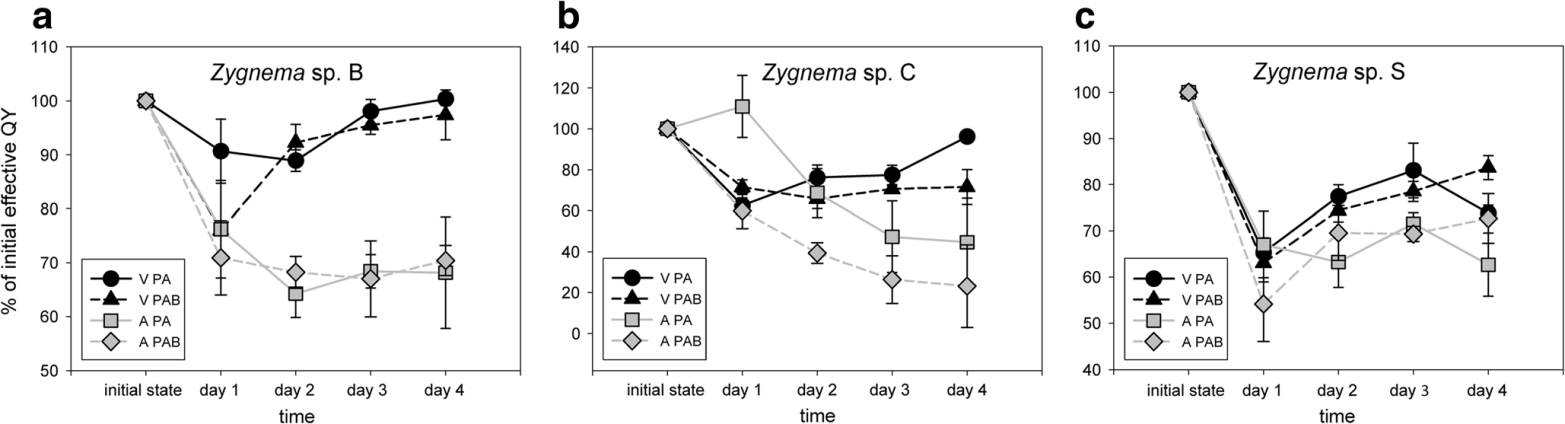
Changes in effective quantum yield (*ϕ*_PSII_) during the experiment. Values relative to the initial values before the UV exposure are shown (mean ± SD, *n* = 3). **a** *Zygnema* sp. B, **b** *Zygnema* sp. C, **c** *Zygnema* sp. S. Black circles: V PA—young vegetative cells, PAR-UV-A (PA) treatment; black triangles: V PAB—young vegetative cells, PAR+UV-A+UV-B (PAB); gray squares: A PA—pre-akinetes, PA; gray rhomb: A PAB—pre-akinetes, PAB

The effective quantum yield (*ϕ*_PSII_) was determined over the whole 74-h course of the experiment, with measurements taken 2 h after initiating the UV-B exposure. Changes compared to untreated samples prior to the experiment were observed (Fig. 1). The mean initial absolute values of *ϕ*_PSII_ were as follows: *Zygnema* sp. B—young vegetative cells 0.55 ± 0.012, pre-akinetes 0.47 ± 0.012; *Zygnema* sp. C—young vegetative cells 0.61 ± 0.02, pre-akinetes 0.3 ± 0.03; and *Zygnema* sp. S—young vegetative cells 0.7 ± 0.012, pre-akinetes 0.66 ± 0.019. These values were set to 100%. In all strains and most treatments, an initial depression of the effective quantum yield was observed (Fig. 1). In *Zygnema* sp. B, the initial value recovered during the experiment in young vegetative cells after both PA and PAB treatments (Fig. 1a). In contrast, pre-akinete cells of *Zygnema* sp. B showed decreases to a much lower value (~ 60–70% of the initial value) and then remained stable throughout the experiment. Vegetative cells of *Zygnema* sp. C showed a similar tendency, whereas the effective quantum yield of pre-akinetes did not recover during the 74-h duration of the experiment (Fig. 1b). Finally, in *Zygnema* sp. S, the pre-akinetes reached 60–70% of their initial quantum yield on day 4, and slightly higher values were measured for young vegetative cells (Fig. 1c). The recovery rate after 74 h was significantly higher in vegetative cells than in pre-akinetes (*p* < 0.0001, Suppl. Table S2; Fig. 1). UV treatment was not significant when analyzed by three-way ANOVA, showing that there was no general pattern in the effect of individual UV treatments on the recovery of the effective quantum yield. This is also supported by a significant interaction of strain and UV treatment (*p* = 0.0014, Suppl. Table S2), proving that the response to UV differed among strains. Therefore, subsequent two-way ANOVA analyses were performed for each strain separately. In *Zygnema* sp. C, PA treatments had significantly better recovery than PAB (*p* = 0.0319, Suppl. Table S2). In contrast, *Zygnema* sp. S showed better recovery in PAB-treated samples (*p* = 0.0058, Suppl. Table S2).

### Photosynthetic pigments and xanthophyll-cycle pigments change upon UV treatment

**Fig. 2:**
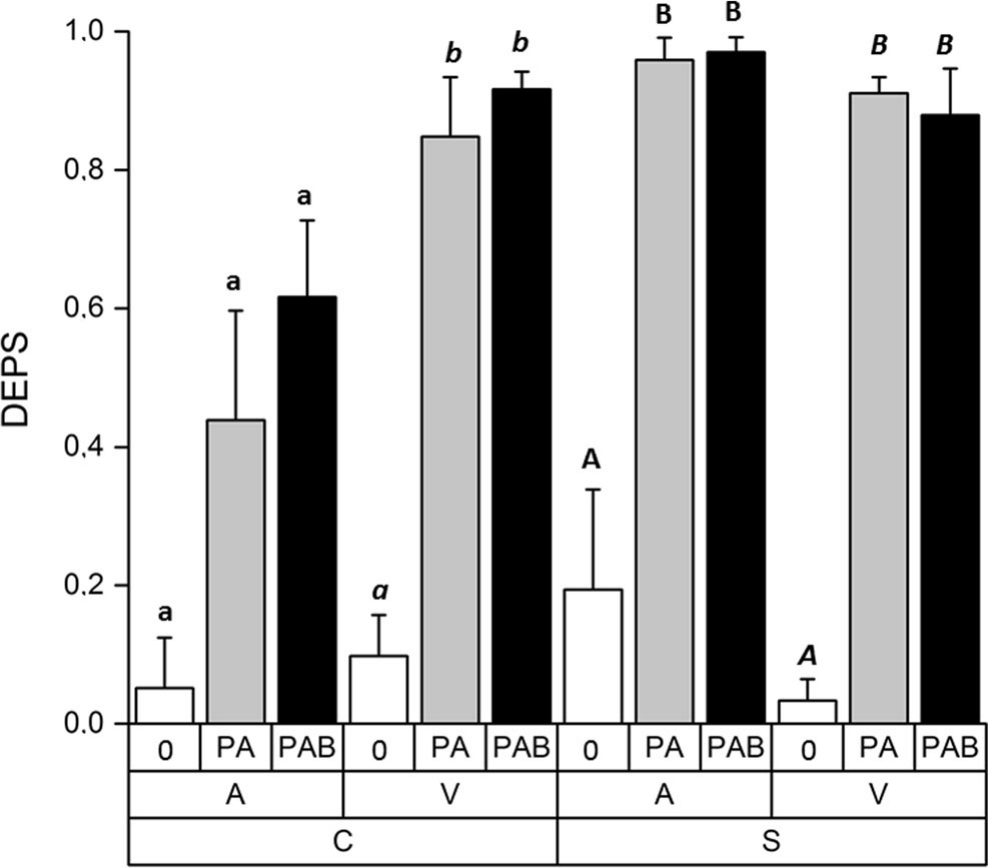
Deepoxidation state—ratio of xanthophyll-cycle pigments antheraxanthin, zeaxanthin, and violaxanthin of *Zygnema* sp. C and *Zygnema* sp. S, (A) pre-akinetes, and (V) vegetative cells either exposed to control condition (0) or PAR+UV-A (PA) or PAR+UV-A+UV-B (PAB). Statistical differences among individual UV treatments (one-way ANOVA, Tukey’s test) are marked with lower-case letters (*Zygnema* sp. C, pre-akinetes), lower-case letters in italics (*Zygnema* sp. C, vegetative cells), upper-case letters (*Zygnema* sp. S, pre-akinetes), or upper-case letters in italics (*Zygnema* sp. S, vegetative cells)

From the total analysis of the primary pigments (Suppl. Fig. S2), we used the xanthophyll-cycle pigments violaxanthin (V), zeaxanthin (Z), and antheraxanthin (A) (Suppl. Fig. S3) to determine the deepoxidation state (DEPS) = (A + Z)/(V + A + Z) of *Zygnema* sp. C and *Zygnema* sp. S. The effects of all factors and their interactions proved significant when tested by three-way ANOVA, indicating that the deepoxidation state of the cultures was influenced by UV treatment, but also the response was different for each strain and culture age. In addition, we found significant differences between the untreated samples and the samples exposed to PA and PAB in all cases, except for pre-akinetes of *Zygnema* sp. C (Fig. 2, Suppl. Table S2). However, no significant differences were found between the two different UV treatments, although the mean values were higher in the PAB treatments in most cases (Fig. 2).

### UV-absorbing phenolic compounds increase as a consequence of UV treatment

**Fig. 3:**
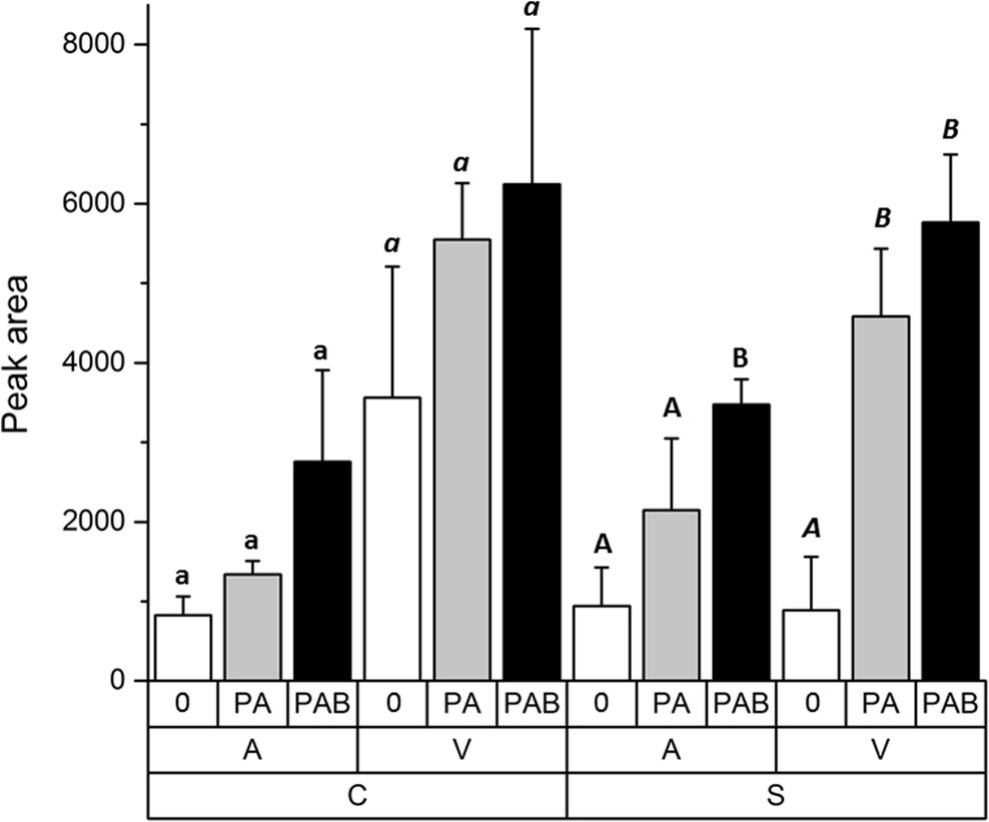
UV-absorbing phenolic compounds, illustrated as peak areas in *Zygnema* sp. C (C, left) and *Zygnema* sp. S (S, right). Pre-akinetes (A) are shown at the left side and vegetative cells (V) at the right side. The different treatments are indicated as follows: untreated control (0), PAR+UV-A (PA), and PAR+UV-A+UV-B (PAB). Statistical differences among individual UV treatments (one-way ANOVA, Tukey’s test) are marked with lower-case letters (*Zygnema* sp. C, pre-akinetes), lower-case letters in italics (*Zygnema* sp. C, vegetative cells), upper-case letters (*Zygnema* sp. S, pre-akinetes), or upper-case letters in italics (*Zygnema* sp. S, vegetative cells)

The effects of both culture age and UV treatment on the content of phenolics were shown to be significant when tested by three-way ANOVA (Table S2, Fig. 3). Both strains shared the same pattern of response to UV: In general, the content of UV-absorbing compounds was higher in vegetative cells than in pre-akinetes (*p* < 0.0001) and there was a tendency towards elevated mean phenolic contents after PA and PAB treatment. However, these changes were not statistically significant in *Zygnema* sp. C when analyzed separately by one-way ANOVA. In *Zygnema* sp. S, phenolics increased significantly after PA and PAB treatment in vegetative cells and after PAB treatment in pre-akinetes compared to untreated samples (Table S2, Fig. 3). This indicated that particularly in *Zygnema* sp. S, PA- and PAB-induced changes in UV-absorbing phenolic compounds, with retention times (RT) of 15.4, 24.8, and 26.1 min (Suppl. Fig. S4). These peaks, while having absorption maxima around 280 nm, were also absorbing in the UV-A range. All other phenolic substances (20 compounds), which had only a single absorption maximum at 280 nm (e.g., the peak at RT 23.4 min, shown in Suppl. Fig. S4), were excluded from further analysis. These compounds are probably precursors or intermediates but contribute only slightly in the biologically important waveband.

### Light microscopy shows differences between vegetative cells and pre-akinetes

**Fig. 4:**
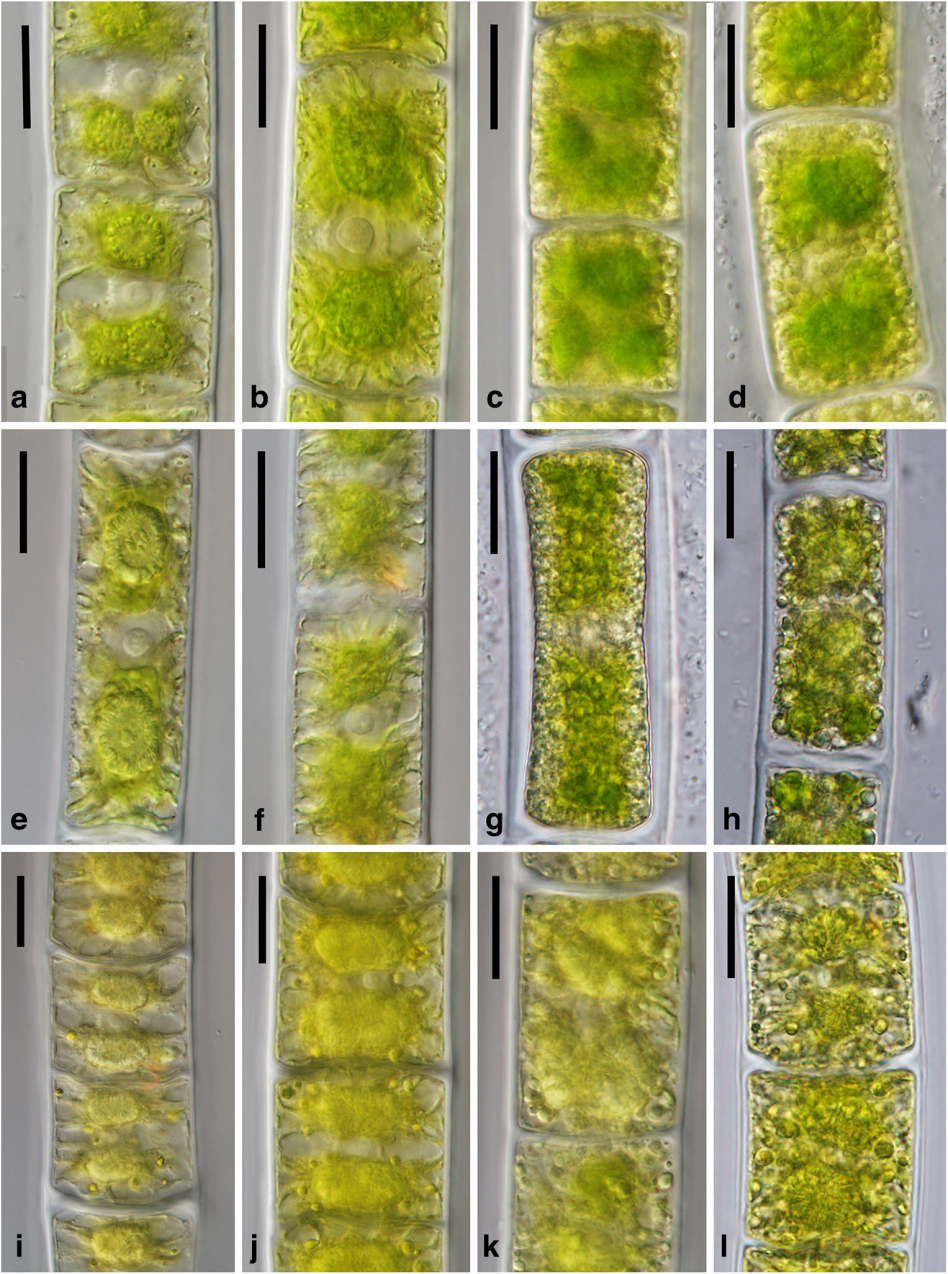
Light micrographs of *Zygnema* cells after exposure to the experimental treatment. *Zygnema* sp. B (**a**–**d**): **a** young cells after PAR+UV-A (PA), **b** young cells after PAR+UV-A+UV-B (PAB), **c** pre-akinetes after PA, **d** pre-akinetes after PAB. *Zygnema* sp. C (**e**–**h**): **e** young cells after PA, **f** young cells after PAB, **g** pre-akinetes after PA, **h** pre-akinetes after PAB. *Zygnema* sp. S (**i**–**l**): **i** young cells after PA, **j** young cells after PAB, **k** pre-akinetes after PA, **l** pre-akinetes after PAB. Scale bars 20 μm

UV treatment had no visible effect on cellular morphology observed under the light microscope (Fig. 4). Young cells of all strains were highly vacuolated, their chloroplasts had numerous lobes protruding towards the cell periphery, and large nuclei were easily visible in the central part of the cells (Fig. 4a–b, e–f, i–j). Cytoplasm of the pre-akinetes appeared denser and contained numerous lipid bodies, and chloroplast lobes were no longer clearly discernible (Fig. 4c–d, g–h, k–l).

### Transmission electron microscopy shows only moderate changes upon addition of UV-B

**Fig. 5:**
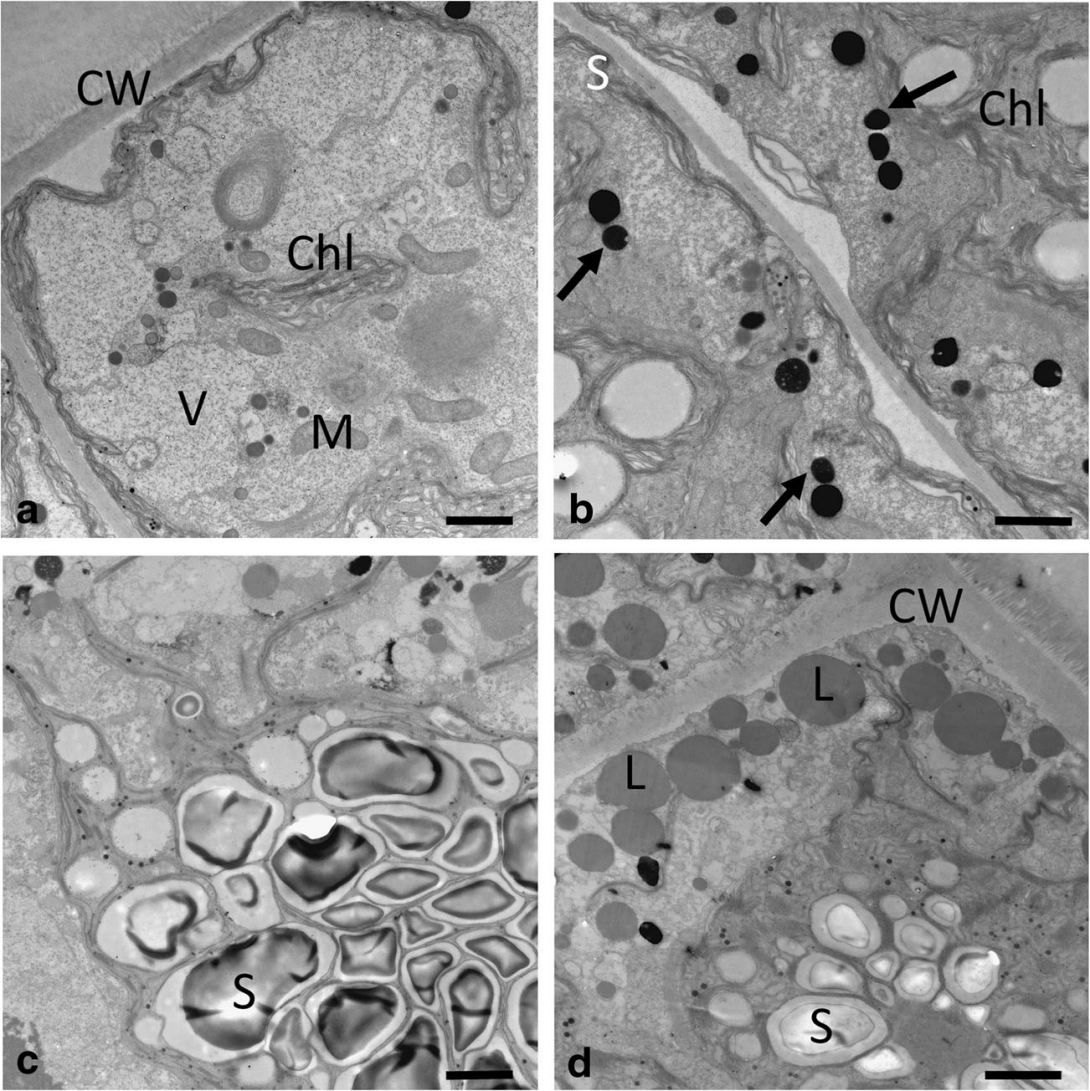
Transmission electron micrographs of *Zygnema* sp. B young vegetative cells (**a**–**c**) and pre-akinete cell (**d**), exposed to **a**, **d** PAR+UV-A (PA) or **b**, **c** PAR+UV-A+UV-B (PAB). **a** Overview of young cell showing extensive vacuolization, and narrow chloroplast lobes, reaching towards the cell periphery. **b** Electron-dense bodies (arrows) are found in the cell periphery. **c** Massive starch accumulations around the pyrenoids. **d** Typical appearance of pre-akinete cells with massive lipid bodies in the cell periphery; the chloroplast shows starch accumulations, and electron-dense bodies are found. *CW* cell wall, *L* lipid body, *M* mitochondrion, *S* starch, *V* vacuole. Bars 2 μm

In young vegetative cells of *Zygnema* sp. B, large accumulations of starch were found under PA exposure, indicating an active metabolism (Suppl. Fig. S5a); the cells showed a high degree of vacuolization and narrow chloroplast lobes (Fig. 5a). Under PAB exposure, more electron-dense bodies appeared in the cell periphery (Fig. 5b; Suppl. Fig. S5b). The cells still contained large starch accumulations at the pyrenoids (Fig. 5c). Pre-akinetes of *Zygnema* sp. B contained large accumulations of lipid bodies, particularly in the cell periphery (Fig. 5d); electron-dense bodies were present in PA-treated cells (Fig. 5d, Suppl. Fig. S6a) but were slightly enhanced in PAB-treated cells (Suppl. Fig. S6b).

**Fig. 6:**
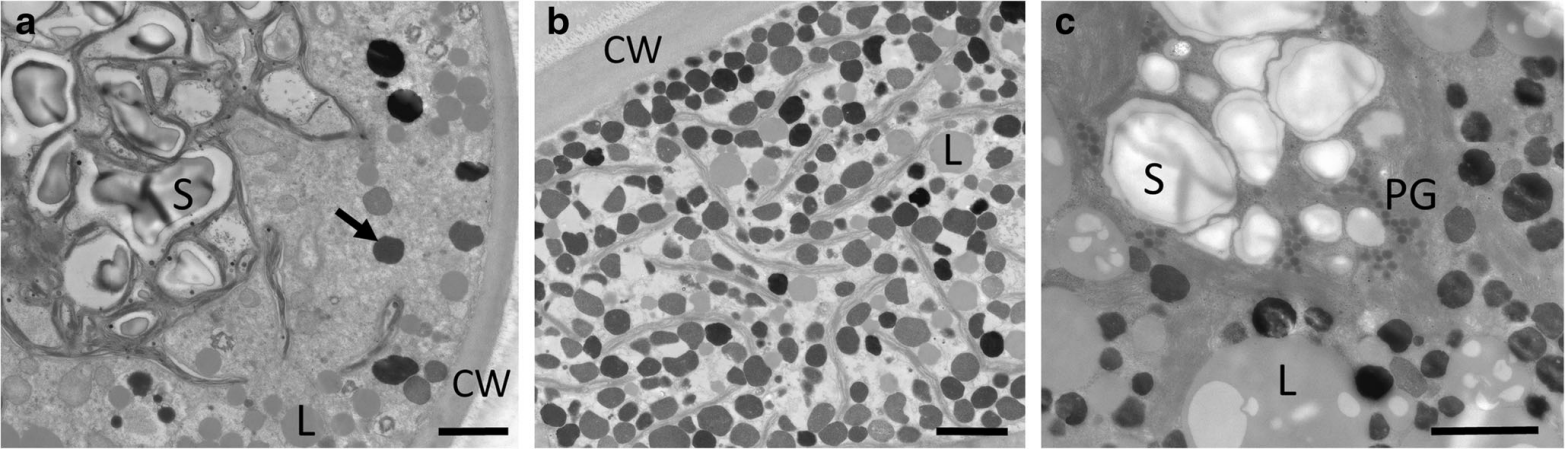
Transmission electron micrographs of *Zygnema* sp. C vegetative cells (**a**, **b**) and pre-akinetes (**c**) exposed to **a** PAR+UV-A (PA) or (**b**, **c**) PAR+UV-A+UV-B (PAB). **a** Numerous starch grains around the pyrenoid; several electron-dense bodies (arrows) and lipid bodies. **b** Cortical section with dense accumulation of electron-dense bodies and lipid bodies. **c** Chloroplast with starch grains and plastoglobules, electron-dense bodies (arrows), and large lipid bodies. *CW* cell wall, *L* lipid body, *PG* plastoglobules, *S* starch. Bars 2 μm

In *Zygnema* sp. C, electron-dense bodies were found in vegetative cells under PA treatment (Fig. 6a) and were sometimes massive under PAB treatment (Fig. 6b). This massive accumulation of electron-dense bodies was not observed in all cells, but a general tendency of increasing occurrence of these structures under PAB treatment, when compared to PA in young cells of *Zygnema* sp. C, was obvious (Suppl. Fig. S5c, d). Pre-akinetes of *Zygnema* sp. C showed an accumulation of lipid bodies, starch grains, and abundant electron-dense bodies, particularly in PAB-treated cells (Fig. 6c). Comparison between PA- and PAB-treated pre-akinetes, however, showed that electron-dense bodies were present in both (Suppl. Fig. S6c, d).

**Fig. 7:**
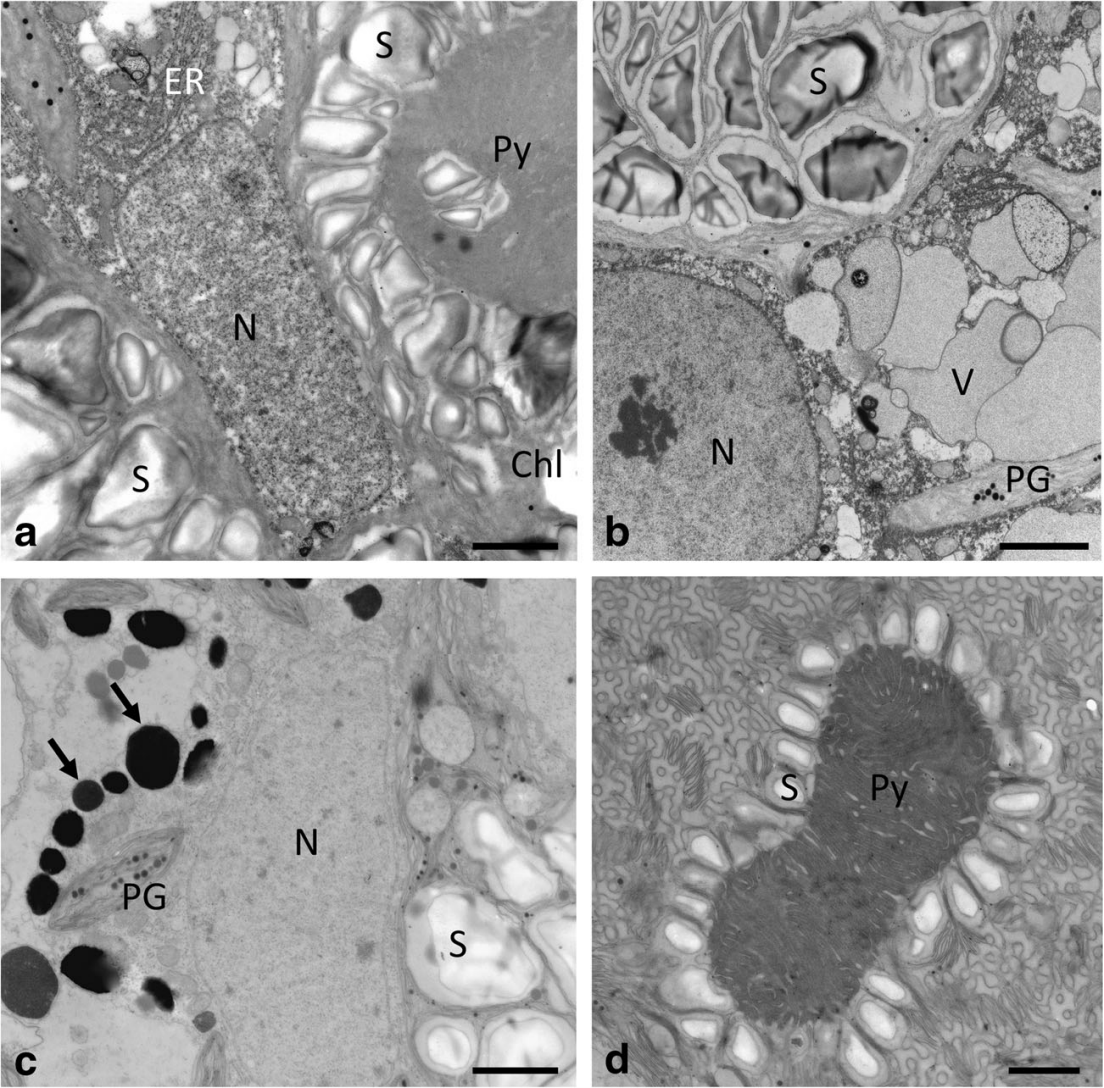
Transmission electron micrographs of *Zygnema* sp. S vegetative cells (**a**, **b**) and pre-akinetes (**c**, **d**). Cells were exposed either to **a** PAR+UV-A (PA) or (**b**–**d**) to PAR+UV-A+UV-B (PAB). **a** Central nucleus surrounded by two chloroplasts with prominent pyrenoids, surrounded by numerous starch grains, ER close to the nucleus. **b** Nucleus with starch-filled chloroplast and individual vacuoles; chloroplast lobes contain plastoglobules. **c** Central area with nucleus, starch grains in the chloroplast, and electron-dense bodies (arrows) and numerous plastoglobules. **d** Pyrenoid surrounded by a single layer of starch grains, thylakoid membranes arranged in a cubic structure. *Chl* chloroplast, *ER* endoplasmatic reticulum, *N* nucleus, *PG* plastoglobules, *Py* pyrenoid, *S* starch. Bars 2 μm

*Zygnema* sp. S had massive starch accumulations around the pyrenoids in young vegetative cells exposed to PA and PAB (Fig. 7a, b). Around the nucleus, dense accumulations of endoplasmic reticulum were observed in PA- and PAB-treated vegetative *Zygnema* sp. S cells (Fig. 7a, b). The high degree of vacuolization of these vegetative cells is illustrated in Fig. 7b and Suppl. Fig. S5e. Electron-dense bodies occurred in both PA- and PAB-treated cells (Suppl. Fig. S5e, f). Electron-dense bodies were found in pre-akinete cells of PAB-treated cells (Fig. 7c), but they were also observed in PA-treated cells (Suppl. Fig. S6e). These cells contained numerous starch grains and lipid bodies (Fig. 7c). The pyrenoids were surrounded by starch grains, and the thylakoid membranes appeared wrinkled (Fig. 7d).

### Metabolomic analysis

**Fig. 8:**
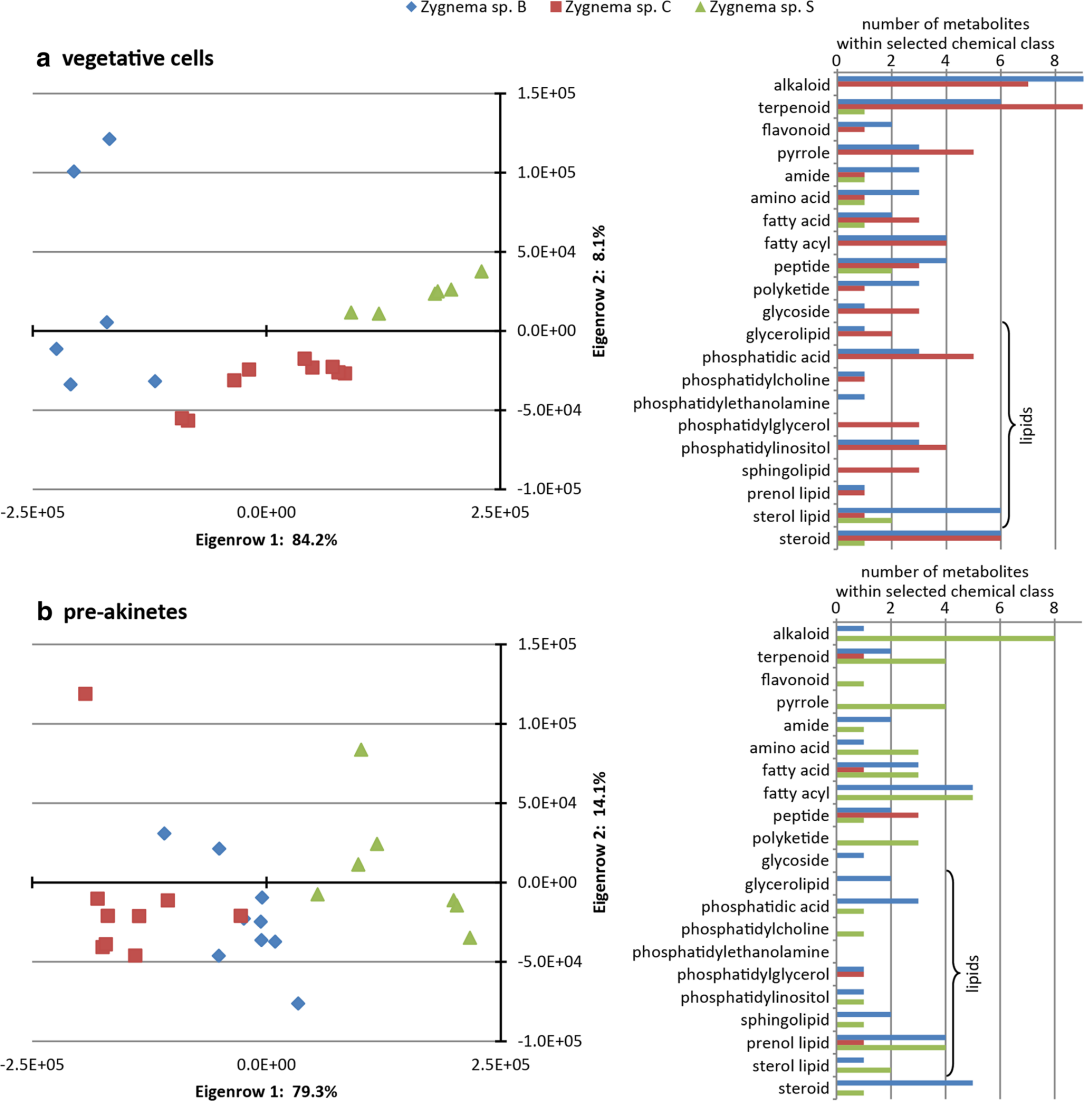
PCA analysis of metabolomic data of young vegetative cells (**a**) and pre-akinetes (**b**). Selected chemical classes driving the separation of *Zygnema* sp. strains within vegetative cells (**a**) and pre-akinetes (**b**) are listed on the right side. The different *Zygnema* strains are indicated by colors: *blue*: *Zygnema* sp. B, *red*: *Zygnema* sp. C, *green*: *Zygnema* sp. S

The UHPLC-qToF-MS analyses revealed a total of 617 molecular masses in the whole set of differently treated *Zygnema* strains. Masses were statistically evaluated for correlations according to UV treatments, culture ages, and strain types. N-Way ANOVA analyses with significance values of *p* < 0.06 defined the data set as non-significant but indicated an association of the applied factors. PCAs were performed to confirm this indicated trend of the metabolomics data. The results showed no differences when all samples were compared. Hence, data were divided into subsets of single *Zygnema* strains and vegetative cells and pre-akinetes, respectively. The correlations thus obtained again indicated no separation of the various UV treatments, but showed a clear trend of *Zygnema* strains of vegetative cells or pre-akinetes (Fig. 8a, b).

Three hundred eighty-four molecular masses, which were responsible for the separation of *Zygnema* spp. in PCAs, were extracted and aligned with chemical databases, i.e., Kyoto Encyclopedia of Genes and Genomes (KEGG), Human Metabolome Database (HMDB), LipidMaps, MetaCyc, KNApSAcK, and PubChem, which yielded around 200 assigned features. Most of these metabolites were classified as alkaloids, steroids, terpenoids, pyrroles, and phospholipids. Figure 8a depicts the number of metabolites in selected chemical classes, related to *Zygnema* spp. B, C, and S, respectively.

Metabolite compositions in vegetative cells of *Zygnema* sp. B and C were very similar, whereas fewer metabolites from selected chemical classes were detected in *Zygnema* sp. S (Fig. 8a). Compared with pre-akinetes (Fig. 8b), high amounts of phospholipid species were found in vegetative cells. The *Zygnema* sp. S pre-akinetes were separated from the Arctic and Antarctic strains based on the higher contents of alkaloids, polyketides, and pyrroles, which indicated ongoing metabolite production in pre-akinetes.

## Discussion

The present study investigated the effects of realistically simulated photosynthetically active radiation (PAR 400 μmol photons m^−2^ s^−1^) in combination with UV-A (PA) or enhanced UV-B (PAB), on three *Zygnema* strains from different geographic regions (Arctic, Antarctic, and temperate). The habitat characteristics for the polar strains were very similar; they grew as hydroterrestrial mats in shallow pools exposed to permanent radiation under polar day conditions (Pichrtová et al. 2014). The temperate strain was exposed to long day conditions during summer season (Herburger et al. 2015), comparable to the experimentally applied 16:8-h light cycle. From each strain, young vegetative cultures and pre-akinetes were investigated. Three-way ANOVA analysis revealed significant differences for the effect of culture age in all physiological parameters tested. Due to their active metabolism, young cells could adjust to the experimental conditions much better by increasing the production of protective substances. The effect of strain was significant in the analyses of effective quantum yield (*ϕ*_PSII_) and deepoxidation state (DEPS) of xanthophyll-cycle pigments. Additionally, the metabolomics approach allowed a clear separation among the strains, when young vegetative cells and pre-akinetes were analyzed separately; however, this approach could not detect effects of the UV treatments.

### Photophysiology suggests good adaptation to experimental UV simulation

Young vegetative cells of all strains recovered their initial values of the effective quantum yield (*ϕ*_PSII_) much better than pre-akinete cells during the course of the experiment. In *Zygnema* sp. C, the initial values of *ϕ*_PSII_ recovered significantly better in PA-treated cells; this effect was reversed in *Zygnema* sp. S, where the PAB-treated cells showed better performance. Similarly, Stamenković and Hanelt (2014) observed an ameliorating effect of UV-B at 21 °C in the tropical *Cosmarium beatum*, as concluded from higher rates of recovery of maximum quantum yield after moderate UV-B treatment. We can conclude that the UV treatments applied here did not drastically change the photophysiological properties of PS II, indicating a still-active physiological performance.

In contrast, negative effects on the *F*_V_/*F*_M_ as well as on *ϕ*_PSII_ were detected upon short-term treatment (6 h) with 1.4 W m^−2^ UV-B in young cultures of an Antarctic *Zygnema* sp. isolate (Prieto-Amador 2016). The observations by Pichrtová et al. (2013) also showed a significant decrease of *F*_V_/*F*_M_, at least in two strains after experimental UV exposure, suggesting that an initial effect on the photosynthetic apparatus in fact occurs.

In vegetative cells of both the Antarctic *Zygnema* sp. C and the temperate *Zygnema* sp. S, a statistically significant elevation of the deepoxidation state of the xanthophyll-cycle pigments was found under PA and PAB exposure, compared to untreated controls. Note that we compared the initial values of samples that were taken directly from the standard culture conditions (0 under low PAR of approx. ~ 38 μmol photons m^−2^ s^−1^), with the sun simulator-incubated samples that were exposed to PA or PAB, both at PAR of 400 μmol photons m^−2^ s^−1^. There was, however, no significant difference between PA and PAB, suggesting that the addition of UV-B was not driving the change. This agrees with earlier findings in *Zygnema* sp., where the UV treatment did not provoke an increase in the deepoxidation state of the xanthophyll-cycle pigments in *Zygnema* spp. E and G, while an increase in the deepoxidation state was found in *Zygnema* sp. B (Pichrtová et al. 2013). Recently, the xanthophyll-cycle turnover was perturbed in an Arctic *Zygnema* sp. by the use of dithiotreitol (DTT), an inhibitor of the violaxanthin deepoxidation (Kakkou et al. 2016). This resulted in a slight increase in chlorophyll fluorescence in the time interval 0 to 0.2 s (J and I chlorophyll fluorescence levels), indicating the importance of the natural rapid conversion of violaxantin into zeaxanthin. In *Cosmarium* sp., xanthophyll-cycle pigments correspond to those of high-light-adapted plants and algae (Stamenković et al. 2014a). Exceptionally, an Arctic isolate (*Cosmarium crenatum* var. *boldtianum*) showed an incomplete violaxanthin cycle, leading to the accumulation of antheraxanthin during high light stress (Stamenković et al. 2014a). In the present study, we also observed reduced values of DEPS in pre-akinetes of the Antarctic strain *Zygnema* sp. C, compared to young cells or the temperate strain. This agrees nicely with the drastically reduced *ϕ*_PSII_ acclimation capacities (~ 20–40% of the initial value) in pre-akinetes of *Zygnema* sp. C.

### Changes in phenolic compounds

Changes in UV-AB-absorbing phenolic compounds as a consequence of UV treatments were found significant when analyzed by three-way ANOVA. This accords well with previous findings, where with a predominantly UV-A treatment, an increase of similar phenolic compounds was observed in Arctic and Antarctic strains of *Zygnema* (Pichrtová et al. 2013).

The HPLC method used in the present study was slightly different from the previously used method (Pichrtová et al. 2013); however, all the major phenolic peaks were found, with similar absorption characteristics. Based on the spectral characteristics, for analysis of phenolic compounds, we considered only peaks with absorption in the UV-A and UV-B range. In young cells of the temperate *Zygnema* sp. S, a significant increase in UV-absorbing phenolic compounds was observed in the PA- and PAB-exposed samples, but in pre-akinetes only in PAB-exposed samples, compared to untreated samples (*p* < 0.05). The significant increase in young cells might be explained by their generally higher metabolic activity. In *Zygnema* sp. C, untreated young vegetative cells already contained high levels of phenolic compounds compared to pre-akinetes, suggesting a constitutive protection mechanism already available under standard culture conditions. The observation that pre-akinetes contained smaller amounts of phenolics compared to young vegetative material might be due to the cell volume being mostly filled with lipids (Pichrtová et al. 2016b), while the phenolics detected are water-soluble. These observations do not support the hypothesis that pre-akinetes are better protected against UV irradiation. In the *Zygnema* strains investigated here, no visible coloration deriving from phenolic derivatives was observed in the light micrographs. However, a detailed chemical characterization of these compounds in *Zygnema* is still lacking.

### Metabolomics allowed separation between strains

Metabolic analysis could not detect an influence of the UV treatments on *Zygnema* sp. strains. The results confirmed that substantial peculiarities of vegetative cells and pre-akinetes dominate metabolic differentiation. A detailed analysis of the metabolites detected in vegetative cells and pre-akinetes, respectively, showed a distinct separation of *Zygnema* sp. strains and indicated changes in their activity at both stages of culture. Vegetative cells of the strains of polar origin (*Zygnema* spp. B and C) were found to be more similar in their metabolite composition (e.g., alkaloids, terpenoids, steroids, pyrroles, and phospholipids) than those in the temperate strain *Zygnema* sp. S. Several of these metabolite classes were found in *Zygnema* sp. S only in the pre-akinete stage, suggesting that they synthesize these compounds later. This interesting observation could possibly point to a geographic attribution, where the temperate strain has a longer growing season in which to synthesize certain compounds. These observations, however, remain to be investigated in more detail in future studies.

### Structural alterations due to UV treatment

The light microscopy observations showed clear differences between young and pre-akinete cells, but no changes could be attributed to the respective UV treatment.

Some indications of stress protection were observed in the ultrastructural investigations in the present study, i.e., (1) electron-dense bodies in the cytoplasm and (2) cubic membranes in the chloroplast. The most prominent structures that have been attributed to UV protection were the electron-dense bodies (Holzinger et al. 2009; Pichrtová et al. 2013). These structures were previously described as “inclusions” in beginning akinetes (McLean and Pessoney 1971), and they have been found in field samples of an Arctic strain (Holzinger et al. 2009). Pichrtová et al. (2013) speculated that these bodies, with a diameter of 400–600 nm, contain phenolics. Here we showed that they could be found basically in all treatments, but there was a tendency of accumulation of these electron-dense bodies in PAB-treated cells, which was illustrated, e.g., in *Zygnema* sp. C (Fig. 6b), where massive accumulations were found in some of the young cells. This observation would concord nicely with the increase of phenolic compounds in young vegetative cells of *Zygnema* sp. C as detected by the HPLC approach. However, we still cannot provide evidence for the chemical nature of these compartments, only that they are highly reactive with osmium tetroxide, leading to the electron-dense appearance.

Cubic membranes, as shown in *Zygnema* sp. S to occur upon PAB treatment (Fig. 7d), have been reported previously in *Zygnema* (e.g., McLean and Pessoney 1970; Zhan et al. 2017). These cubic membranes are attributed to a stress-defense reaction, as they usually occur after high light exposure (Zhan et al. 2017). However, the studies by McLean and Pessoney (1970) and Zhan et al. (2017) used approximately the same light intensities. Recently, cubic membranes have been considered as an antioxidant-defense system (Deng and Almsherqi 2015). They were also observed in the desmid *Cosmarium* after high-temperature treatment (Stamenković et al. 2014b).

In general, the ultrastructure of all *Zygnema* strains showed an intact appearance in both PA- and PAB-treated cells, concording with earlier results (Holzinger et al. 2009; Pichrtová et al. 2013). The massive occurrence of lipid bodies in pre-akinete cells has been reported repeatedly (McLean and Pessoney 1971; Pichrtová et al. 2014, 2016b) and was also found in the present study. These lipid bodies are formed during prolonged culture and have never been observed in young vegetative cells (e.g., Bakker and Lokhorst 1987; Pichrtová et al. 2013). Lipid bodies are, together with starch accumulations, ideal for energy storage, but are not involved in UV tolerance.

## Conclusion

Against our hypothesis that pre-akinetes could tolerate UV radiation better, the results indicated that particularly young vegetative *Zygnema* sp. cells are well protected and able to acclimate to conditions of increased PAB. This can be concluded from the significantly better recovery rate of the *ϕ*_PSII_ values during the 74-h experiment. The young vegetative cells had higher initial *ϕ*_PSII_ values than the pre-akinetes, as previously reported (Pichrtová et al. 2014). These observations are supported by the significantly higher amount of UV-absorbing phenolic compounds in young vegetative cells. In young *Zygnema* sp. S, PA and PAB treatment induced a significant increase of phenolic compounds, compared to untreated cells. Moreover, the deepoxidation state of the xanthophyll-cycle pigments increased significantly upon PA and PAB treatments, suggesting a good light protection in general. This was also supported by ultrastructural observations of protective structures such as electron-dense bodies and cubic membranes in the chloroplast.

The strains were well separated by the metabolomics approach (the metabolites of the Arctic and Antarctic strains were more similar to each other) and showed differences in physiological performance (the Antarctic strain had significantly lower *ϕ*_PSII_ values after PAB, while the temperate strain recovered better under PAB). An association of these observations with the geographic origin of the strains is possible, but must be interpreted critically, as only one strain per region was investigated.

## Electronic supplementary material

ESM 1 (DOCX 4246 kb)

ESM 2 (DOCX 12 kb)

ESM 3 (XLSX 18 kb)
