# Supplementary material for: Arctic, Antarctic, and temperate green algae Zygnema spp. under UV-B stress: vegetative cells perform better than pre-akinetes
Source: Protoplasma. 2018 Feb 22;255(4):1239–52. doi: 10.1007/s00709-018-1225-1 (PMC5994220; doi:10.1007/s00709-018-1225-1)
Supplement: Supplementary file 1 — (DOCX 4246 kb) [file 709_2018_1225_MOESM1_ESM.docx]

Supplementary Figures:


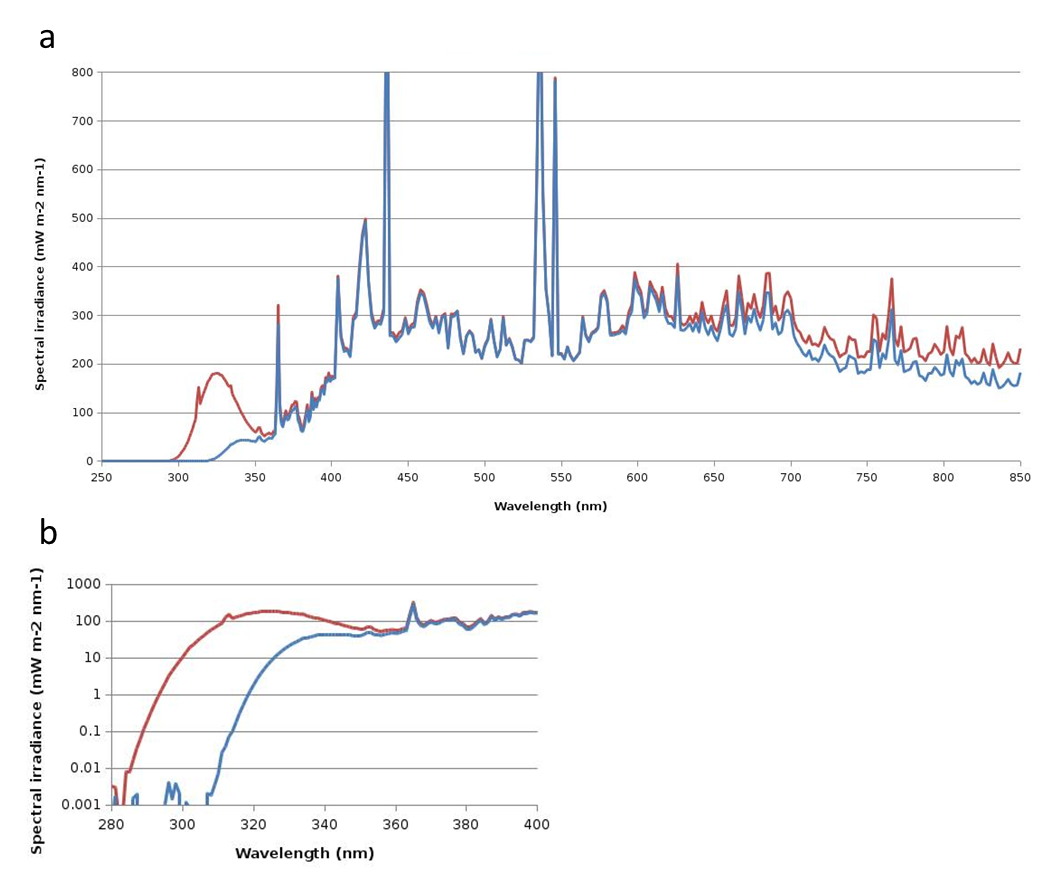


Suppl. Fig. S1 Spectral properties of the two irradiation conditions measured from 250 to 850 nm including UV-B and UV-A radiation (red line), UV-A radiation only (blue line). **a** linear scale, **b** UV range from 280 to 400 nm in logarithmic scale..


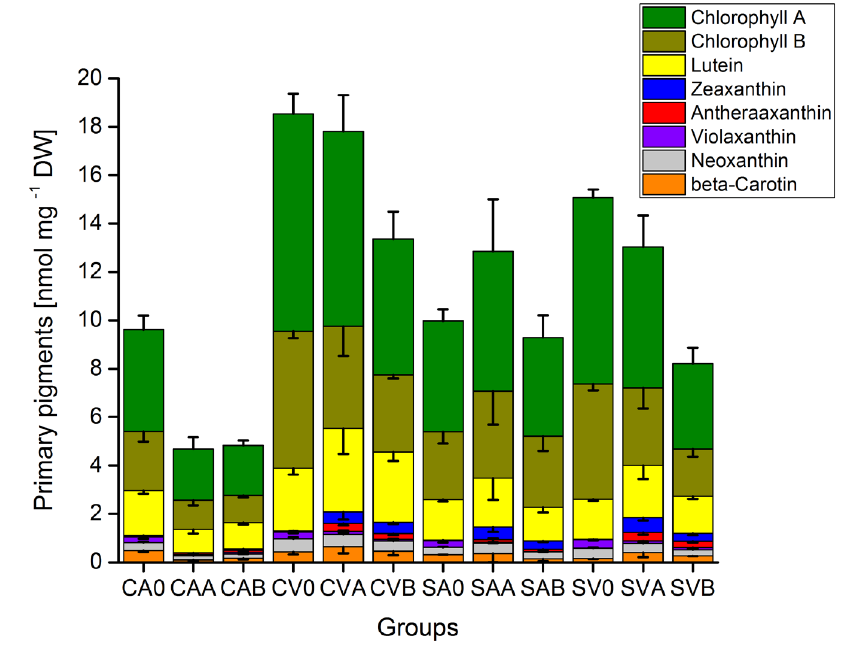


Suppl. Fig. S2 Absolute values of primary pigments in *Zygenma* C and *Zygnema* S.


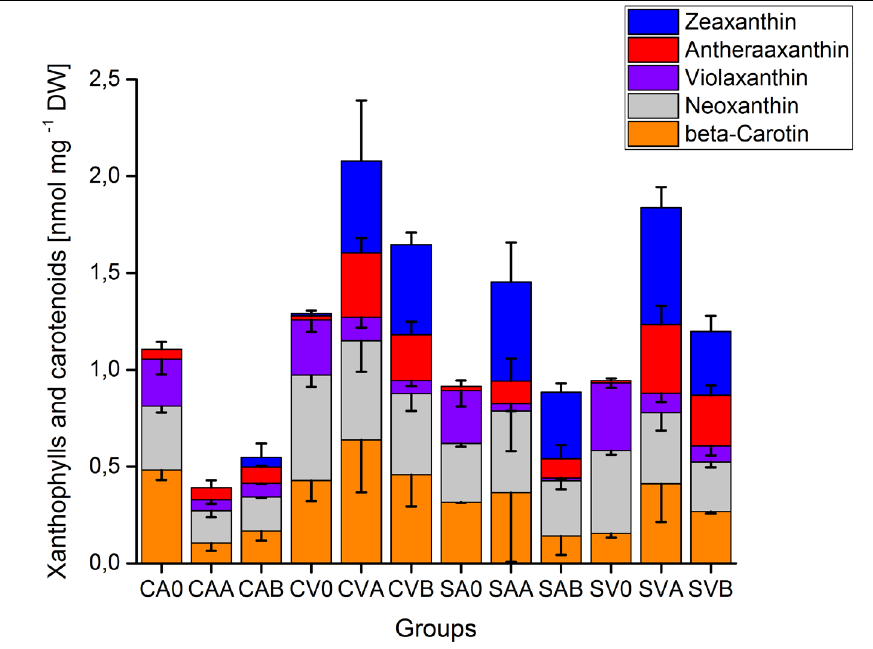


Suppl. Fig. S3 Xanthophyll cycle pigments (Zea, Anth, Viola) as well as Neoxanthin and beta-Carotin. D


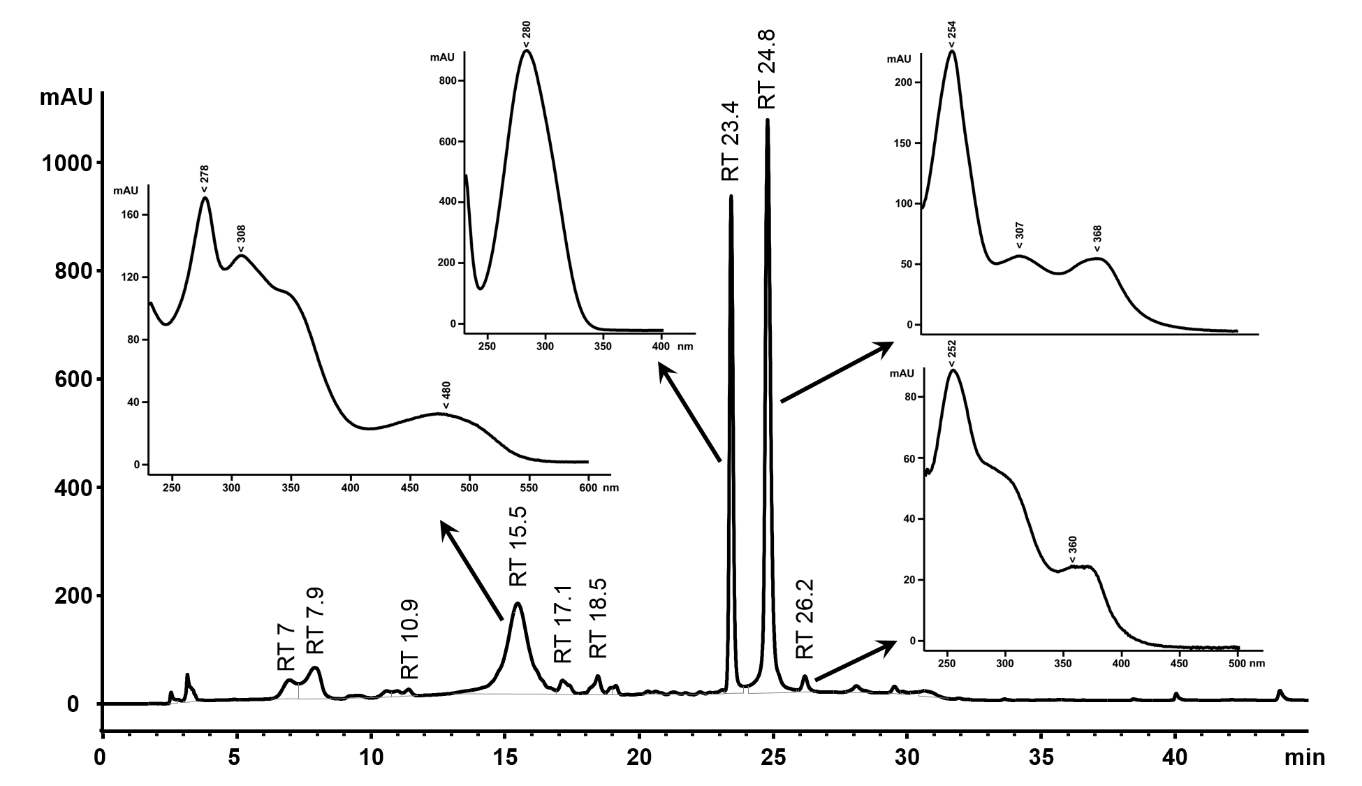


Suppl. Fig. S4 Representative HPLC chromatogram, displaying the spectra at RT 15.4 min, 24.8 min, 26.1 min.


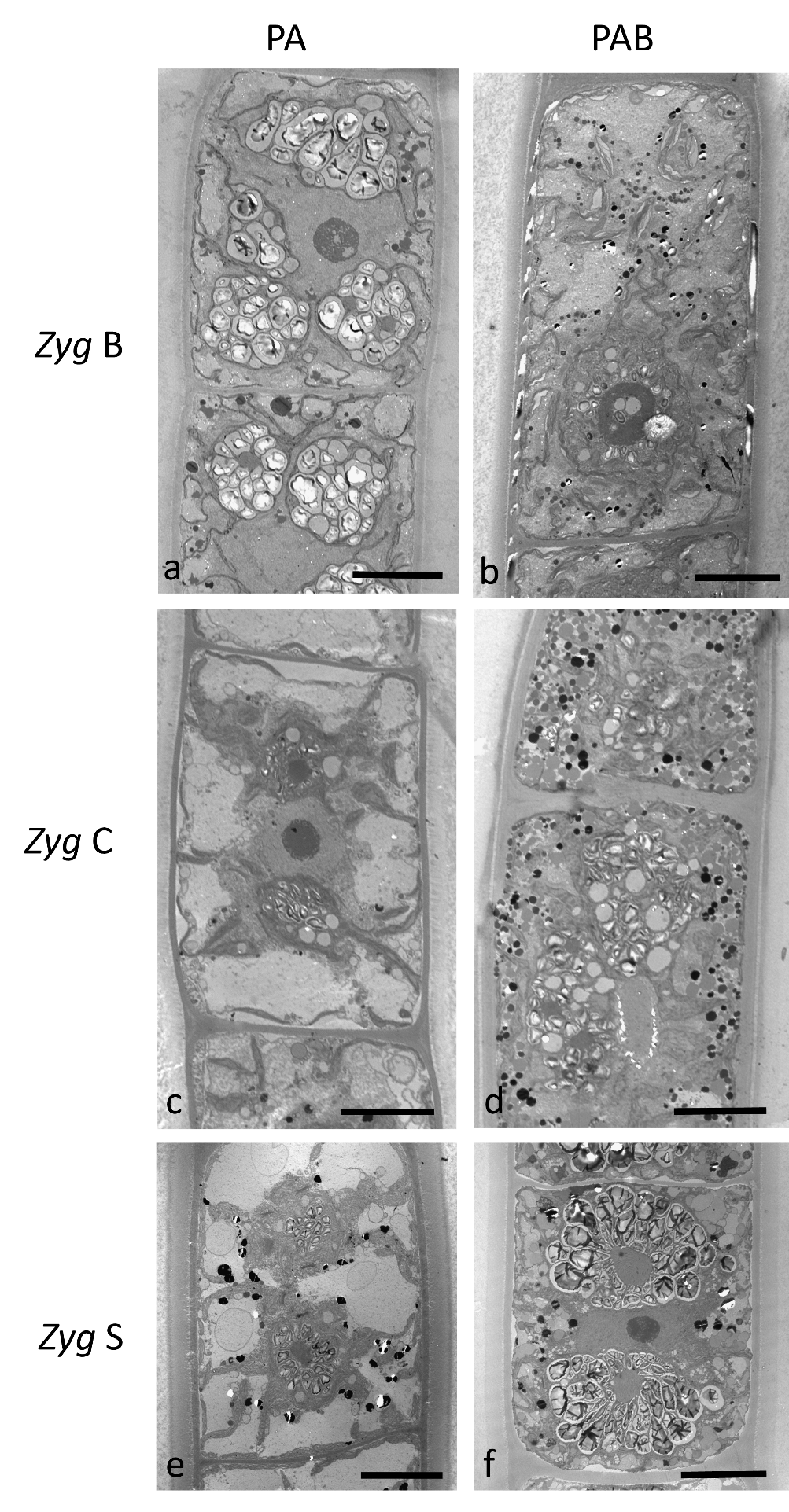


Suppl. Fig. S5 Transmission electron micrographs of vegetative cells of (a-b) *Zygnema* B, (c-d) *Zygnema* C and (e-f) *Zygnema* S, exposed either to UV A (a, c, e) or UV AB (b, d, f). The images give overviews of representative cells. Abbreviations: N nucleus, Chl chloroplast, V vacuole. Bars 10 µm


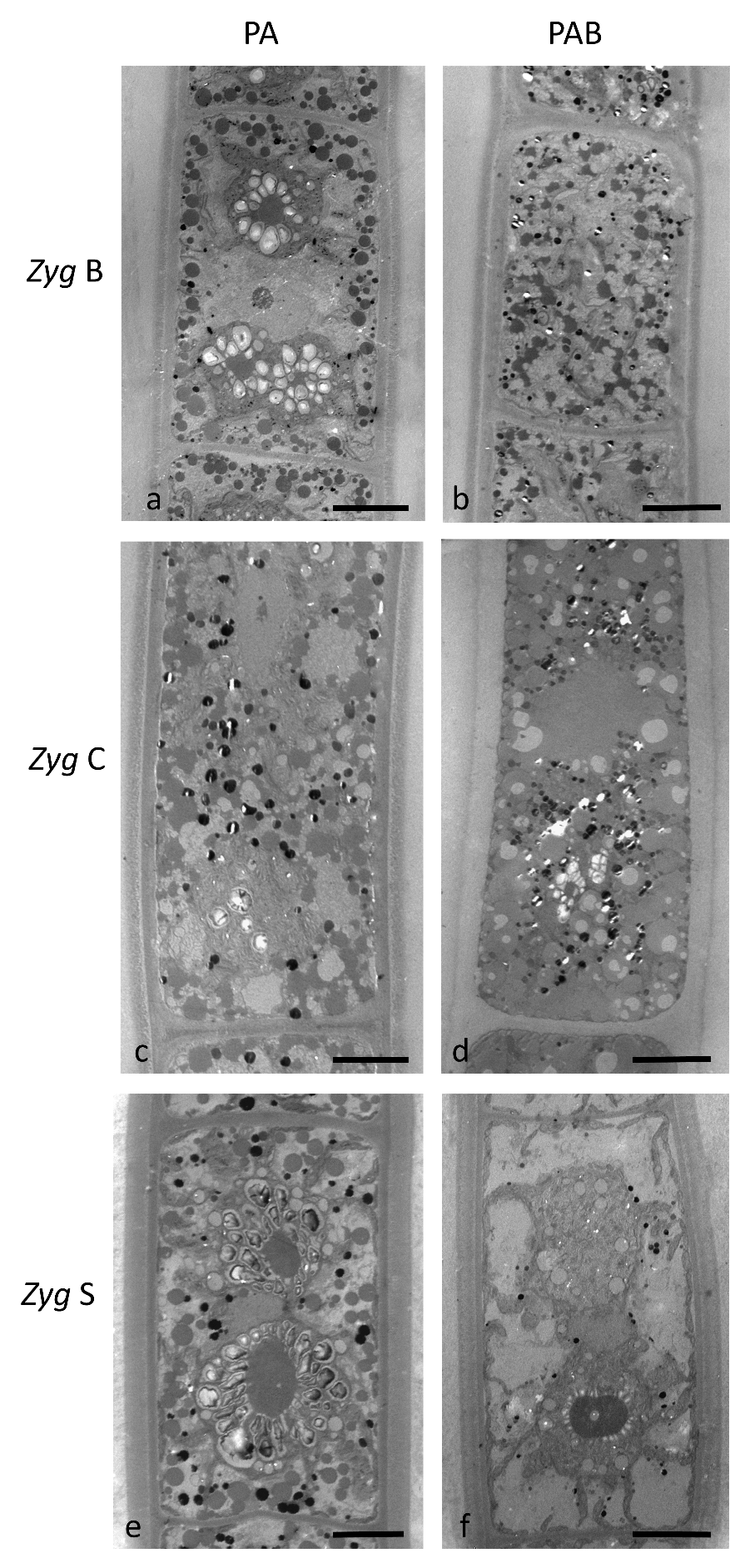


Suppl. Fig. S6 Transmission electron micrographs of pre-akinete cells of (a-b) *Zygnema* B, (c-d) *Zygnema* C and (e-f) *Zygnema* S, exposed either to UV A (a, c, e) or UV AB (b, d, f). The images give overviews of representative cells. Abbreviations: N nucleus, Chl chloroplast, V vacuole. Bars 10 µm
