# Supplementary material for: Arctic, Antarctic, and temperate green algae Zygnema spp. under UV-B stress: vegetative cells perform better than pre-akinetes
Source: Protoplasma. 2018 Feb 22;255(4):1239–52. doi: 10.1007/s00709-018-1225-1 (PMC5994220; doi:10.1007/s00709-018-1225-1)
Supplement: Supplementary file 2 — (DOCX 12 kb) [file 709_2018_1225_MOESM2_ESM.docx]

Suppl. Table S1. Parameters of UHPLC-qToF-MS analysis

| **Parameters RP-UHPLC** | **Value** |
| --- | --- |
| Injection volume | 5 µL |
| Injection type | Partial Loop |
| Weak wash volume | 3000 µL |
| Strong wash volume | 2500 µL |
| Temperature Autosampler | 4 °C |
| Pre-aspirate volume | 1 µL |
| Temperature colum | 40 °C |
|  |  |
| **Parameters qToF-MS detection** | **Value** |
| ***ESI source*** |  |
| Ionization mode | positive |
| End plate offset | - 500 V |
| Capillary voltage | 4500 V |
| Nebulizer pressure | 2.0 Bar |
| Dry gas flow rate | 10.0 I/min |
| Dry gas temperature | 200°C |
| ***Quadrupole*** |  |
| Ion energy | 3.0 eV |
| Low mass | 100 m/z |
| ***Collision Cell*** |  |
| Collision energy | 8.0 eV |
| Collision RF | 500.0 Vpp |
| ***Ion Cooler*** |  |
| Transfer time | 75 μs |
| Ion cooler RF | 75.0 Vpp |
| Pre pulse storage | 10.0 μs |
| ***Others*** |  |
| Mass range | 100 m/z – 1500 m/z |
| Spectra rate | 2.0 Hz |
